# Oxidative Stress and Pathogenesis in Malaria

**DOI:** 10.3389/fcimb.2021.768182

**Published:** 2021-11-30

**Authors:** Marilyn Vasquez, Marisol Zuniga, Ana Rodriguez

**Affiliations:** Department of Microbiology, New York University Grossman School of Medicine, New York, NY, United States

**Keywords:** malaria, *Plasmodium falciparum*, *Plasmodium vivax*, oxidative stress, reactive oxygen species, oxidation, pathogenesis, cerebral malaria

## Abstract

Malaria is a highly inflammatory and oxidative disease. The production of reactive oxygen species by host phagocytes is an essential component of the host response to *Plasmodium* infection. Moreover, host oxidative enzymes, such as xanthine oxidase, are upregulated in malaria patients. Although increased production of reactive oxygen species contributes to the clearance of the parasite, excessive amounts of these free radicals can mediate inflammation and cause extensive damage to host cells and tissues, probably contributing to severe pathologies. *Plasmodium* has a variety of antioxidant enzymes that allow it to survive amidst this oxidative onslaught. However, parasitic degradation of hemoglobin within the infected red blood cell generates free heme, which is released at the end of the replication cycle, further aggravating the oxidative burden on the host and possibly contributing to the severity of life-threatening malarial complications. Additionally, the highly inflammatory response to malaria contributes to exacerbate the oxidative response. In this review, we discuss host and parasite-derived sources of oxidative stress that may promote severe disease in *P. falciparum* infection. Therapeutics that restore and maintain oxidative balance in malaria patients may be useful in preventing lethal complications of this disease.

## Introduction

Oxidative stress is caused by reactive oxygen or nitrogen atoms that have unpaired electrons in their outer shell. They are called reactive oxygen species (ROS) or reactive nitrogen species (RNS) and are commonly produced in cells. These radicals are oxidants that can damage cellular components, but are also involved in essential cellular processes, such as intracellular signaling and the oxidative burst in innate immune cells (Liguori et al., 2018).

Oxidative stress has been related to aging and to a variety of diseases including diabetes, cancer and cardiovascular complications, based on a general hypothesis that molecular damages induced by ROS and RNS result in the functional impairments that underlie aging and the aforementioned diseases (Liguori et al., 2018). However, the role of oxidative stress in infectious diseases is more complex, since it contributes to the elimination of invading pathogens, but also causes molecular damage in the host. It is well known that infections frequently induce high levels of ROS and RNS that are formed as part of the inflammatory response, and also as a consequence of organ damage and metabolic changes induced by infection (Pohanka, 2013).

There is a tight relation between inflammation and oxidative stress during any infection (Nathan and Cunningham-Bussel, 2013). It is well known that innate immune cells recognize pathogens and respond by triggering strong inflammatory responses. Innate immune cells phagocytose these pathogens and attempt to eliminate them by rapidly increasing the production of ROS in their phagosomes in a mechanism called oxidative or respiratory burst. ROS produced during the oxidative burst are also released extracellularly, contributing to the increase of the oxidative state in the infected host (Thomas, 2017). Inflammatory and oxidative pathways are linked in immune cells through the transcription factor NF-kB, which is activated by inflammatory mediators and, in turn, can activate pro-oxidant genes in these cells (Lingappan, 2018).

Conversely, oxidative stress can induce inflammation, since ROS regulate the inflammatory response in immune cells through the activation of NF-kB, which results in the secretion of inflammatory cytokines (Lingappan, 2018). ROS also serves as the first signal for the activation of the inflammasome (Ty et al., 2019), further contributing to the inflammatory response.

Malaria is a highly inflammatory and oxidative disease. During the blood stage of infection, the level of oxidative stress in plasma is frequently measured by determining the concentration of malondialdehyde (MDA), a lipid peroxide which is formed as a consequence of oxidation of unsaturated lipids and reflects the levels of free radicals in the circulation (Ayala et al., 2014). This method has allowed for the determination of the levels of oxidative stress in plasma samples from malaria patients. Results indicate that oxidative stress is higher in malaria patients, caused by either *Plasmodium falciparum* or *P. vivax* infection, compared to healthy controls (Das and Nanda, 1999; Pabon et al., 2003; Yazar et al., 2004; Prasannachandra et al., 2006; Tiyong Ifoue et al., 2009; Bilgin et al., 2012; Narsaria et al., 2012). Additionally, high levels of oxidative stress were found in monkeys infected with *P. knowlesi* (Srivastava et al., 1992) and mice infected with *P. berghei, P. yoelii* or *P. chabaudi* (Nneji et al., 2013; Scaccabarozzi et al., 2018), indicating that oxidative stress is a generalized phenomenon in *Plasmodium* infections.

The increase in oxidative stress observed in malaria patients infected with either *P. falciparum* or *P. vivax* infections is often coupled with a decrease of anti-oxidant levels (**Table 1**). This decrease in both enzymatic and non-enzymatic antioxidants during malaria evidences the loss of the homeostatic balance between free radicals and antioxidant capacity that is maintained in healthy tissues.

**Table 1:** Malaria patients have significantly increased lipid peroxidation and decreased levels of antioxidants.

*Plasmodium* species	Lipid peroxidation	Antioxidants	Study
*P. falciparum*	↑	↓ Catalase ↓ Glutathione ↓ Tocopherol ↓Ascorbate	(Das and Nanda, 1999)
*P. vivax*	↑	↓ Catalase ↓ Glutathione ↓ Superoxide dismutase ↓ Glutathione peroxidase ↓ Ascorbate ↓ Vitamin A	(Erel et al., 1997)
*P. falciparum* *& P. vivax*	↑	↓ Tocopherol	(Prasannachandra et al., 2006)
*P. falciparum & P. vivax*	↑	↓ Catalase	(Pabon et al., 2003)
*P. falciparum*		↓ Vitamin E ↓ Vitamin A	(Delmas-Beauvieux et al., 1995)
*P. vivax*	↑	↓ Superoxide dismutase ↓ Glutathione peroxidase	(Bilgin et al., 2012)
*P. falciparum*	↑	↓ Ascorbate	(Das et al., 1993)
*P. falciparum* & *P. vivax*	↑	↓ Superoxide dismutase ↓ Vitamin E	(Kulkarni et al., 2003)

During the blood stage of *Plasmodium* infection, a relation between oxidative stress and inflammation is evidenced by ROS-induced activation of macrophages (Ty et al., 2019) and dendritic cells (Gotz et al., 2019), which results in the secretion of inflammatory cytokines. Studies *in vitro* showed that ROS not only activates classical cytokine secretion in these cells, but also provided a first essential signal necessary for inflammasome activation, with the parasite *P. falciparum* providing the second one (Ty et al., 2019). *Plasmodium* infections in mice show a gradual increase in both inflammatory cytokines and oxidative stress in different organs (Gosavi et al., 2016). In *P. falciparum* patients, the fact that cytokine levels and oxidative stress both increase with disease severity (Abdullahi et al., 2020), and the finding of a direct correlation between inflammatory cytokines and ROS levels in patient plasma, suggest a link between inflammation and oxidative stress in malaria (Ty et al., 2019).

During the initial liver stage of malaria, the parasite is susceptible to oxidative stress which decreases parasite survival within the hepatocytes. Interestingly, a high-fat diet which increased the levels of oxidative stress was found to be highly protective of liver stage malaria infection (Zuzarte-Luis et al., 2017). Conversely, high levels of heme-oxygenase-1 (HO-1), which has a strong antioxidant activity, promote *Plasmodium* liver infection (Epiphanio et al., 2008). Since HO-1 is part of the inflammatory response induced in the host by *Plasmodium* infection, the parasite would ultimately benefit from this response because of reduced levels of oxidative stress that would otherwise impair its growth. This constitutes a perfect example of the close relation between the inflammatory and oxidative responses during infection and the important consequences that are derived for the host.

## Sources of Oxidative Stress During Malaria

**Figure 1:**
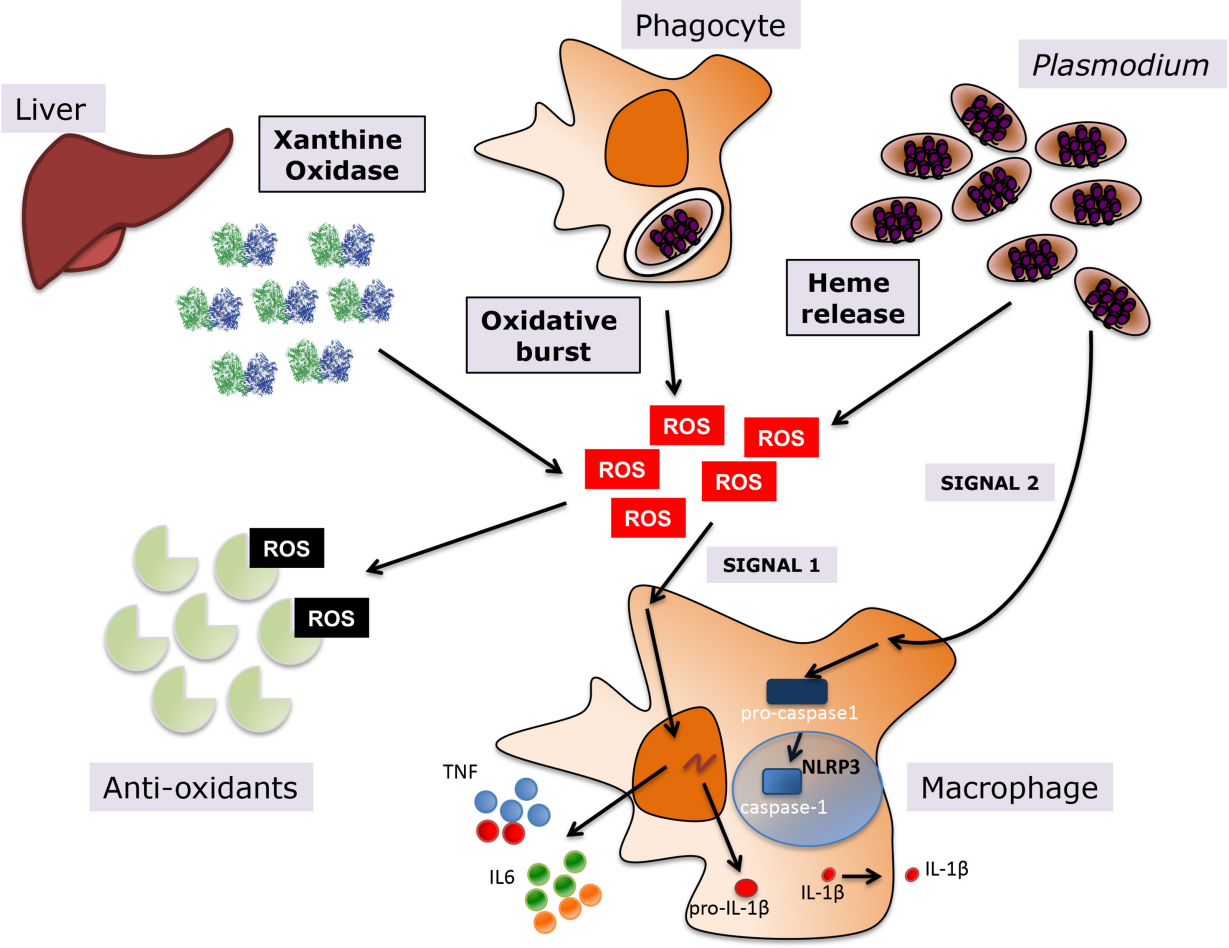
Oxidative stress during *Plasmodium *infection. Various sources contribute to the oxidative environment during malaria, including upregulation of host enzymes such as XO, the oxidative burst in macrophages upon phagocytosis of infected erythrocytes, and heme release from hemoglobin degradation in host infected erythrocytes. A balance between levels of anti-oxidants in the human host and the generation of ROS determines the levels of oxidative stress. ROS promote inflammation in malaria, leading to the activation of macrophages and the subsequent release of pro-inflammatory cytokines, such as TNF and IL6 among others, but also inflammasome-dependent IL1-β, where ROS provide priming signal 1 and *P. falciparum* the activating signal 2.

During the blood stage of malaria, there are different sources of host-derived oxidative stress that are either a direct result of *Plasmodium* infection of erythrocytes, such as heme, or a consequence of the host response to infection, which includes systemic upregulation of host oxidative enzymes and the phagocytic oxidative burst (**Figure 1**). Additionally, anti-malarial treatments are frequently contribute to oxidative stress, since their mechanism of action is mediated by oxidative killing of the parasite.

### Heme

Heme is a source of oxidative stress that affects both the parasite and the host during malaria. *Plasmodium* parasites consume hemoglobin within infected erythrocytes as a source of amino acids (Becker et al., 2004). The breakdown of hemoglobin results in the release of heme, which can produce reactive oxygen species *via* its iron atom. Heme iron can promote the production of the hydroxyl radical *via* the Fenton reaction, which can induce damage to the parasite and the host (Percario et al., 2012). To counteract the adverse effects of heme, *Plasmodium* converts heme into hemozoin, an intracellular aggregate where heme molecules are interlinked, forming a crystal structure. Hemozoin does not induce oxidative damage to the host or parasite.

However, previous reports observed that not all heme molecules are successfully crystallized into hemozoin within the infected erythrocyte (Becker et al., 2004), meaning that free heme that has not been converted to hemozoin can cause damage to host cells and tissues once it has been released. Heme can also be released *via* hemolysis of uninfected red blood cells, which also occurs during *Plasmodium* infection (Haldar and Mohandas, 2009). Therefore, lysis of both infected and uninfected erythrocytes are potential contributors to oxidative stress in malaria.

### Xanthine Oxidase

Another source of oxidative stress during *Plasmodium* infection is the host oxidative enzyme xanthine oxidase (XO). XO generates reactive oxygen species such as superoxide (O_2_·-) and hydrogen peroxide (H_2_O_2_) as it breaks down hypoxanthine to xanthine and ultimately, to uric acid (Battelli et al., 2016). Elevated levels of this enzyme have been reported in both mouse (Tubaro et al., 1980) and human infections (Iwalokun et al., 2006; Ty et al., 2019) with *Plasmodium* parasites. Pediatric patients with severe malaria (Iwalokun et al., 2006) and adult patients with cerebral malaria (Ty et al., 2019) have higher levels of XO in circulation compared to patients with uncomplicated malaria, supporting a pathogenic role for this enzyme in exacerbating severity of disease during *Plasmodium* infection. XO also contributes to inflammation in malaria, since XO-derived extracellular ROS, in combination with lysates of *P. falciparum-*infected red blood cells (iRBCs), activate monocyte-derived macrophages *in vitro*, leading to the production of the inflammatory cytokine IL-1β and chemokines IL-8, CCL5, and CCL2 (Ty et al., 2019). This enzyme also promotes maturation and cytokine secretion in dendritic cells incubated with *P. falciparum-*iRBCs (Gotz et al., 2019). Moreover, XO augmented the ability of *P. falciparum* – activated dendritic cells to induce T cell proliferation, contributing to both dendritic cell and T cell responses to *Plasmodium* infection (Gotz et al., 2019).

Despite its role in promoting inflammation in malaria, there are reports suggesting a protective role for XO in the context of *Plasmodium* infection. XO interferes with the growth of *in vitro* cultures *P. falciparum*, given that it consumes hypoxanthine, which the parasite needs to synthesize purines (Berman et al., 1991). Moreover, XO-generated ROS have also been implicated as detrimental agents for the growth of *Plasmodium* parasites. Cultures of *P. yoelii* that are pre-treated with XO and subsequently injected into mice are unable to replicate effectively within a murine host, as indicated by the lower parasitemia in these animals compared to that of other animals that were challenged with untreated *P. yoelii*. However, pre-incubation of *P. yoelii* cultures with XO in the presence of the antioxidant enzyme catalase restored the virulence of the parasite, indicating that XO-produced ROS interfered with its survival within the host (Dockrell and Playfair, 1984).

### Phagocytic Oxidative Burst

The production of ROS is a crucial part of the host response to various infectious agents, including *Plasmodium*. The innate immune system of the host uses the oxidative burst to eliminate invading pathogens. This mechanism is triggered in immune cells such as neutrophils and macrophages after phagocytosis of microbial pathogens. Cells activate nicotinamide adenine dinucleotide phosphate reduced (NADPH) oxidase to produce high concentrations of ROS in the phagosome that result in the death of the microbe (Forman and Torres, 2002).

Exposure of human neutrophils and monocytes to *P. falciparum* merozoites and secreted antigens *in vitro* triggers the oxidative burst in these cells, leading to increased production of ROS (Kharazmi et al., 1987). While the oxidative burst is meant to curb the replication and survival of the parasite, it has also been shown that phagocytosis of *Plasmodium* antigens can be detrimental for the oxidative capacity of phagocytic cells, since macrophages that phagocytosed purified hemozoin had diminished NADPH oxidase activity (Schwarzer and Arese, 1996). A study conducted with neutrophils isolated from Gambian children with *P. falciparum* malaria showed that these cells have diminished oxidative burst capacity compared to neutrophils isolated from healthy controls (Cunnington et al., 2012), lending further support to the hypothesis that the oxidative burst can be compromised during *Plasmodium* infection. Overall, downregulation of the phagocytic oxidative burst may be beneficial in that it decreases oxidative stress for the host, but its inhibition would also facilitate the growth and survival of the parasite.

### Oxidative Stress Induced by Anti-Malarial Drugs

Given that *Plasmodium* parasites are sensitive to ROS-mediated damage, it is not surprising that various antimalarial treatments exploit this feature of the parasite to limit its growth within human hosts. Quinolones, including chloroquine and amodiaquine, act by inhibiting the conversion of free heme to hemozoin within the infected erythrocyte, effectively increasing oxidative stress for *Plasmodium* parasites (Kavishe et al., 2017). Although the principal mechanism of chloroquine resistance is based on the ability of the parasites to prevent the drug from accumulating in their digestive vacuole, an increase in parasite antioxidant capacity may contribute to resistance. Notably, it has been shown the chloroquine-resistant strains of human (*P. falciparum)* and rodent (*P. yoelii*, and *P. berghei)* malaria have increased activity of the antioxidant enzyme glutathione-S-transferase compared to chloroquine-sensitive strains of the same species (Srivastava et al., 1999), which may help these parasites counteract the oxidative burden caused by the drug.

Another major family of anti-malarial drugs are the artemisinins, which include dihydroartemisinin and artesunate (Kavishe et al., 2017). These drugs also exert their anti-parasitic effects by increasing oxidative stress on the parasite. One of the proposed mechanisms of action for artemisinin postulates that its interaction with iron leads to the production of free radicals (Cui and Su, 2009). Notably, the antioxidant N-acetylcysteine (NAC) can counteract the anti-parasitic effects of artesunate on *P. falciparum*, further supporting the hypothesis that these drugs act by promoting the production of free radicals that are harmful to the parasite (Arreesrisom et al., 2007).

While increasing oxidative stress is an effective mechanism of action for parasite elimination, it may also have a negative impact on the host. Artesunate-induced oxidative damage was observed *in vitro* in human cellular DNA (Berdelle et al., 2011) and in treated mice (Singh et al., 2015). Moreover, a study showed that malaria patients treated with anti-malarial drugs had higher levels of lipid peroxidation and lower levels of anti-oxidants compared to non-treated patients (Akanbi et al., 2010), suggesting that anti-malarial drugs increase oxidative stress in the host. Another report proposed using antioxidants, such as quercetin, along with chloroquine to offset the oxidative stress and toxic effects caused by this drug (Kumar Mishra et al., 2013).

## Oxidative Stress and the Pathogenesis of Malaria

It is well documented that the levels of oxidative stress increase with severity in *P. vivax* (Sibmooh et al., 2004; Ray et al., 2016; Aqeel et al., 2021) and *P. falciparum* (Das and Nanda, 1999; Greve et al., 2000; Narsaria et al., 2012) infections, suggesting that oxidation may contribute to the development of complications in malaria. Although specific mechanisms have not been characterized, it is possible that ROS contributes to disease severity directly through detrimental effects of oxidative stress on the tissues or indirectly through the increase in the inflammatory response (Hemmer et al., 2005; Taoufiq et al., 2006).

It is known that oxidative stress induces lipid peroxidation in the surface of erythrocytes, reducing the deformability in these cells (Becker et al., 2004). Notably, *in vitro* cultures of *P. falciparum* induced lipid peroxidation of both infected and uninfected erythrocytes (Omodeo-Sale et al., 2003). In *P. falciparum* malaria patients, reduced erythrocyte deformability which was mostly observed in uninfected erythrocytes, correlated strongly with death (Dondorp et al., 1997). Since lipid peroxidation reduces erythrocyte deformability, and this has been linked to increased mortality of adults and children with malaria (Dondorp et al., 2002), it has been proposed that oxidative stress contributes to severe malaria pathogenesis through this mechanism. Possible negative consequences of erythrocyte rigidity are microcirculation obstruction and anemia caused by the increased splenic removal of uninfected rigid erythrocytes (Becker et al., 2004).

The role of oxidative stress in cerebral malaria has been studied extensively in mouse models. In mice genetically modified to express a recombinant indicator of oxidation, it was observed that oxidative stress in the brain is dependent on the development of experimental cerebral malaria (Imai et al., 2014). A positive effect of antioxidant treatments in protection of mice from the development of cerebral malaria and cognitive impairments indicates a role for oxidative stress in the pathogenesis of this complication (Thumwood et al., 1989; Kremsner et al., 1991; Reis et al., 2010; Imai et al., 2014; Nyariki et al., 2019). However, mice deficient in the production of ROS by NADPH oxidase, a key oxidative enzyme involved in the phagocytic burst, developed cerebral malaria at similar rate as wild-type mice (Sanni et al., 1999). This lack of difference in the development of pathology may reflect that there are different factors contributing to oxidative stress in the host. The knock-out of one enzyme is probably not sufficient to reduce significantly the overall oxidative burden that the host faces during infection.

The role of heme-induced oxidative stress in malaria pathogenesis has been validated in different *in vivo* studies using knockout mice lacking the gene for HO-1. HO-1 breaks down heme into biliverdin and carbon monoxide (CO), which are antioxidants (Silva et al., 2020), and iron, which can be bound by the iron-binding protein ferritin (Balla et al., 2005). Expression of HO-1 in the context of *Plasmodium* infection is important in the prevention of cerebral malaria in mice (Pamplona et al., 2007). In this study, BALB/c mice challenged with *P. berghei* increased expression of HO-1 at the mRNA and protein levels and did not develop experimental cerebral malaria. On the other hand, C57BL/6 mice did develop cerebral malaria upon challenge with *P. berghei*, but they did not increase expression of HO-1. Notably, treatment of the infected C57BL/6 mice with cobalt protoporphyrin (CoPPIX), a HO-1 inducer, increased survival and prevented damage to the blood brain barrier (Pamplona et al., 2007). HO-1 expression is also involved in protection against acute kidney injury (Ramos et al., 2019) and acute lung injury (Pereira et al., 2016) that occur in mice during *Plasmodium* infection, indicating that heme may play a role in mediating various malarial complications.

Analysis of oxidative stress in brain histology of patients who died of cerebral malaria showed that HO-1 was predominantly found in the vicinity of vessels and hemorrhages. However a similar pattern was also found in patients who died of other causes, suggesting that the proximity of HO-1 to vessels and hemorrhages may be a consequence, rather than a cause of the hemorrhage. In general, there was no pattern of widespread irreversible cell damage found in these patients, which suggests that oxidative stress is not causing generalized damage to the brain (Medana et al., 2001).

However, studies in patients suggest that heme may play an important role in different malaria complications. A study measuring plasma levels of free heme and hemopexin (a molecule that binds to heme facilitating its degradation) found that the ratio of heme to hemopexin was strongly associated with different malaria complications, including severe anemia, respiratory distress and acute kidney damage, as well as mortality after 6 months (Elphinstone et al., 2016). Additional studies in patients show that the levels of cell free hemoglobin and lipid peroxidation (an indicator of oxidative stress) are associated with acute kidney injury (Plewes et al., 2017), further suggesting a detrimental role of heme-induced oxidation in kidney function. Furthermore, adjunctive treatment with acetaminophen, which inhibits lipid peroxidation induced by free heme, resulted in decreased kidney damage in malaria patients (Plewes et al., 2018). Taken together, these results indicate an important role for heme-induced oxidation in malaria pathogenesis and open a promising path for adjunctive treatment.

It is possible that oxidative stress contributes to infected erythrocyte sequestration in the brain microvasculature by inducing increased expression of endothelial adhesion molecules such as ICAM-1 and CD36 (Becker et al., 2004). This effect may be direct, with oxidative stress potentiating the expression of adhesive receptors (Terada, 2002) or indirect, through the increase in cytokine levels, such as TNF, that strongly increase the expression of adhesive receptors.

It has also been proposed that oxidative stress may promote malaria-induced thrombocytopenia, since there was a negative correlation between the number of platelets and levels of lipid peroxidation in malaria patients (Erel et al., 2001; Araujo et al., 2008).

Several clinical trials have been performed to test the efficacy of the antioxidant N-Acetylcysteine (NAC) as an adjunctive treatment for severe malaria. A pilot study found that NAC induced a faster normalization of lactate levels [an predictor of severity and death (White et al., 1985)] in patients with severe malaria compared to the placebo (Watt et al., 2002), supporting its potential use as an adjunctive treatment for severe malaria patients. However, larger studies showed no improvements in clinical outcomes (Treeprasertsuk et al., 2003; Charunwatthana et al., 2009). It was observed that NAC-treated patients presented with significantly higher levels of parasitemia, which was probably a consequence of a major antagonistic effect of NAC on the antimalarial activity of artesunate, which was being used to treat these patients (Charunwatthana et al., 2009). However, NAC adjunctive treatment had no effect in the levels of oxidative stress detected in treated patients compared to untreated controls, suggesting that this treatment was not effective at decreasing the levels of oxidative stress in patients (Charunwatthana et al., 2009).

Human clinical trials using the antioxidant pentoxifylline as adjunctive treatment for cerebral malaria showed conflicting results ranging from a significant decrease in shortening of comma resolution time and death (Das et al., 2003), to small marginally significant effects (Di Perri et al., 1995), to no effect with a non-significant increase of death in the pentoxifylline-treated group (Lell et al., 2010). Attempts to decrease oxidation during malaria by administering vitamin C, which has anti-oxidative properties, did not result in any measurable changes in cytokine levels or improvement in anemia in *P. vivax* patients (Zen Rahfiludin and Ginandjar, 2013).

In general, the disappointing results of some of the antioxidants in malaria clinical trials may be due to the inability of these treatments to significantly decrease oxidative stress in patients. Since the only trial that measured the levels of oxidative stress in treated patients found no effects in their oxidation levels (Charunwatthana et al., 2009), it remains unknown whether systemically decreasing the levels of oxidative stress in patients would have a beneficial effect in pathogenesis.

## Discussion

The precise role and the mechanisms of oxidative stress in malaria pathogenesis are still not well defined. On one hand, there is abundant evidence of strong correlations between levels of oxidative stress in malaria patients and disease severity in general disease severity or specific complications. Together with multiple studies in mice treated with antioxidants, the data suggest that oxidative stress is a major contributor to malaria pathogenesis. On the other hand, despite multiple proposed mechanisms, there is no specific demonstrated causal link between oxidative stress and pathogenesis in human malaria.

Clinical trials with antioxidant treatments could provide a causal relation between oxidative stress and malaria pathogenesis in patients. However, it is unclear whether the anti-oxidative treatments used in the trials actually reduced oxidative stress in the patients, leaving unanswered the question of antioxidants efficacy as adjunctive treatment for severe malaria.

There is a need for mechanistic studies that can dissect the effects of oxidative stress in specific pathogenic mechanisms during malaria. Additionally, the relation between inflammation and oxidative stress, two fundamental players in the pathogenesis of infection, is still not fully understood.

Overall, maintaining oxidative balance in the host in the context of *Plasmodium* infection may be beneficial as it could prevent the development of pathologic complications. However, since the oxidative burst in phagocytes and anti-malarial treatments mechanism of action is mediated by inducing oxidative killing of the parasite, the possible interference of antioxidant treatments with parasite elimination must be carefully analyzed before treatment.

## Author Contributions

MV, MZ, and AR wrote the manuscript. All authors contributed to the article and approved the submitted version.

## Funding

MV, MZ, and AR are supported by NIH grants 1R01NS105910 and 1R01HL150145.

## Conflict of Interest

The authors declare that the research was conducted in the absence of any commercial or financial relationships that could be construed as a potential conflict of interest.

## Publisher’s Note

All claims expressed in this article are solely those of the authors and do not necessarily represent those of their affiliated organizations, or those of the publisher, the editors and the reviewers. Any product that may be evaluated in this article, or claim that may be made by its manufacturer, is not guaranteed or endorsed by the publisher.
